# Structural brain imaging biomarkers for predicting seizure recurrence after a first unprovoked seizure

**DOI:** 10.1002/epi4.70236

**Published:** 2026-02-17

**Authors:** Suyi Ooi, Chris Tailby, Heath R. Pardoe, Patrick W. Carney, Moksh Sethi, Jonas Haderlein, Graeme D. Jackson, David N. Vaughan

**Affiliations:** ^1^ The Florey Institute of Neuroscience and Mental Health Melbourne Brain Centre Melbourne Victoria Australia; ^2^ Florey Department of Neuroscience and Mental Health The University of Melbourne Melbourne Victoria Australia; ^3^ Department of Neurology Austin Health Heidelberg Victoria Australia; ^4^ Department of Clinical Neuropsychology Austin Health Heidelberg Victoria Australia; ^5^ Eastern Health Clinical School Monash University Box Hill Victoria Australia; ^6^ Department of Neurology Northern Health Epping Victoria Australia; ^7^ Faculty of Medicine, Dentistry and Health Sciences The University of Melbourne Melbourne Victoria Australia

**Keywords:** Epilepsy prediction, first unprovoked seizure, machine learning, seizure recurrence, structural MRI

## Abstract

**Objectives:**

Predicting seizure recurrence following a first unprovoked seizure (FUS) remains a significant clinical challenge, especially when routine clinical magnetic resonance imaging (MRI) and EEG do not reveal abnormalities diagnostic of epilepsy. Here, we incorporate quantitative structural MRI‐derived biomarkers into prediction models for seizure recurrence at 12 months and identify brain structural features that are predictive of seizure recurrence.

**Methods:**

We analyzed a retrospective, multicenter cohort of 197 adult patients with FUS, comprising 83 with seizure recurrence and 114 with no seizure recurrence at 12 months. All participants had normal or nondiagnostic MRI and EEG findings. Morphometric features were extracted from clinical 3 T T1‐weighted MRI using FreeSurfer. Machine learning algorithms were trained on combined imaging and clinical features using nested cross‐validation for model selection. Performance was compared with a logistic regression model based on clinical features only.

**Results:**

The best‐performing model, a support vector machine (SVM) trained on a combination of imaging features and clinical factors, achieved an AUC of 0.65 (95% CI: 0.57–0.73), significantly better than chance (*p* = 0.01 when compared with an AUC of 0.5). In contrast, the logistic regression model trained on clinical factors alone yielded an AUC of 0.57 (95% CI: 0.49–0.65), not statistically different to chance (*p* = 0.28). Direct comparison between the SVM and the logistic regression clinical factor‐only model was not statistically significant (95% CI for the difference in AUC: −0.019 to 0.173, *p* = 0.11). The most important imaging features for prediction were inter‐hemispheric asymmetry of subcortical and cortical gray matter volumes and regional gyral curvatures, particularly in fronto‐parietal and limbic regions.

**Significance:**

Quantitative structural MRI contributes additional information beyond clinical factors for machine learning models predicting seizure recurrence. Changes to cortical folding and gray matter asymmetries in cortical and subcortical regions show potential as prognostic biomarkers of seizure recurrence risk after a FUS.

**Plain Language Summary:**

Identifying individuals who will have another seizure after their first unprovoked seizure is difficult when routine brain scans and EEG appear normal. We developed a tool that combines MRI‐derived markers with clinical information to predict seizure recurrence. Subtle structural differences in the brain, especially asymmetries between left and right hemispheres and changes to cortical folding, were associated with a higher chance of another seizure within a year. This approach has potential in identifying individuals at risk of seizure recurrence earlier.

**Key points**

- Quantitative structural MRI features contribute to seizure recurrence prediction after first unprovoked seizure, when combined with clinical data.
- Increment in performance was driven by imaging features, particularly inter‐hemispheric asymmetry and cortical curvature, while clinical factors remained important.
- Predictive imaging features localized to frontal, parietal, and limbic regions.
- Findings support the potential of imaging and advanced biomarkers to further improve outcome prediction after a first seizure.

## 1 INTRODUCTION

Identifying individuals who will go on to have another seizure after a first unprovoked seizure (FUS) remains a major clinical dilemma. After a FUS, 40%–52% of patients will have seizure recurrence within 2 years.^1^ The occurrence of a second seizure meets the International League Against Epilepsy (ILAE) definition for epilepsy, often leading to commencement of anti‐seizure medication (ASM). Alternatively, the finding of epileptiform abnormalities on EEG or an epileptogenic lesion on brain magnetic resonance imaging (MRI) after a FUS can also be used to make an epilepsy diagnosis.^2^ The unresolved challenge is to identify those who will have a second seizure when routine investigations such as MRI and EEG appear normal. In this highly uncertain situation, a range of clinical factors can inform an increased risk of seizure recurrence.^1^, ^3^, ^4^ However, current seizure prediction models using clinical factors alone have shown modest performance, with area under the receiver operating characteristic curve (AUC) values of 0.55 to 0.60^5^, ^6^ (whereby AUC of 1.0 indicates perfect prediction and 0.5 indicates no predictive ability), limiting their use in clinical practice.

Routine MRI visual assessment at the first seizure often does not aid in prediction of epilepsy as an epileptogenic abnormality is not present in more than 80% of cases,^7^, ^8^ leaving prognostication uncertain. Nevertheless, advances in image acquisition and quantitation have shown structural changes in chronic epilepsy syndromes.^9^, ^10^ These changes include structural alterations affecting cortical and subcortical gray matter (GM) thickness and volume, cortical folding and inter‐hemispheric asymmetry.^11^, ^12^, ^13^ While these findings often relate to chronic epilepsy, it is likely that subtle structural brain changes are present in some individuals who are otherwise “MRI‐negative” at the time of their first seizure, and these changes are meaningfully predictive biomarkers of an ongoing predisposition to seizures.

In this work, we aimed to create a prediction tool for 12‐month seizure recurrence after FUS where routine clinical MRI and EEG show no epileptogenic or epileptiform abnormalities, assessing if T1‐weighted (T1w) MRI features can improve seizure recurrence prediction beyond clinical features alone.

## 2 METHODS

### 2.1 Participants

We assembled a retrospective, multicenter, first seizure cohort (total *n* = 197) from consecutive patients seen at three metropolitan tertiary hospital First Seizure Clinics in Melbourne, Australia (Austin Health – HREC/57333/Austin‐2019, Eastern Health – S23‐019‐57 333, and Northern Health – SSA/57333/NH‐2023), seen between 2016 and 2023 inclusive. These dedicated clinics aim to provide rapid‐access specialist epileptology assessment for individuals presenting with a first suspected seizure.

Inclusion criteria were:

- A diagnosis of FUS as determined by a neurologist subspecializing in epilepsy.
- Patients with 12 months or more clinical follow‐up duration.

Exclusion criteria were:

- Epileptiform EEG leading to a diagnosis of epilepsy, as reported by a neurologist subspecializing in epilepsy.
- Epileptogenic lesion on MRI, as reported by a neuroradiologist, prior or after their first clinic assessment of FUS, leading to a diagnosis of epilepsy. Excluded epileptogenic lesions, defined as those with an established relationship with epileptogenicity, included hippocampal sclerosis, malformations of cortical development, cortical gliosis (from previous stroke, traumatic, encephalitis), cavernomas, and low‐grade epilepsy‐associated tumors (e.g., gangliogliomas).^8^
- Patients started on ASM after the initial diagnosis of FUS based on a clinician‐guided treatment decision, as this may have impacted their seizure recurrence risk compared with ASM‐naïve individuals.

### 2.2 Clinical features

Ten categorical clinical features were collected from the medical record. Demographic variables included age and sex. Index seizure type was expressed using two binary variables, specifically, whether the seizure had focal or tonic–clonic seizure semiology. Focal seizure referred to focal impaired consciousness seizure (FIC) and focal to bilateral tonic–clonic seizure (FBTC). Tonic–clonic seizure included FBTC, and bilateral TCS (BTC) unknown whether focal or generalized. This approach reflects lack of reliable information about seizure onset after many first tonic–clonic seizure presentations, though focal semiology can still be identified when present.^14^ We noted if the seizure arose from sleep, and if co‐existing developmental encephalopathy, a neurodegenerative disorder, and a first‐degree family history of epilepsy were present. We noted the presence or absence of nonepileptogenic abnormalities reported on MRI brain and nonepileptiform (i.e., equivocal) EEG abnormalities (e.g., focal or generalized slowing). Nonepileptogenic MRI abnormalities were defined as lesions without a well‐established epileptogenic association and included findings with unclear or inconsistent links to epileptogenesis (e.g., diffuse or focal brain atrophy, or nonspecific T2‐weighted [T2w] FLAIR white matter hyperintensities greater than expected for age).

See Table S1 for reported nonepileptogenic MRI and nonepileptiform EEG abnormalities.

### 2.3 MRI data

Three tesla (3 T) MRI performed as part of the clinical workup was retrospectively collected. T1w sequences with 3D volumetric and isotropic 0.9 mm^3^ resolution or better, and coronal T2w with ≤1 mm in‐plane resolution perpendicular to the long axis of the hippocampus were selected, in line with ILAE Harmonized Neuroimaging of Epilepsy Structural Sequences (HARNESS)‐MRI protocol.^15^ If available, 3D volumetric and isotropic 0.9 mm^3^ or better FLAIR was collected and used together with T1w imaging during image processing. We excluded scanning sites where there were less than five subjects to enable image harmonization, ultimately including subjects scanned on six different scanners across three health services. See Table S2 for the number of patients from each MRI scanner and acquisition parameters.

#### 2.3.1 Image processing and quality assurance

T1w images underwent cortical reconstruction and volumetric segmentation with the Freesurfer image analysis software (version 7.4.0) image processing.^16^ In brief, this processing includes removal of nonbrain tissue, Talairach transformation, segmentation of the subcortical white matter and deep GM volumetric structures, surface deformation following intensity gradients to determine tissue class borders, and intensity normalization. Following this, the cerebral cortex is parcellated into regions with respect to gyral and sulcal structure, from which features based on the Desikan–Killiany cortical atlas were extracted.^17^ In participants with both T1w and 3D volumetric FLAIR, both sequences were used jointly to improve the determination of the pial surface.^18^ Following this, additional Freesurfer processing was performed for cortical local gyrification index (LGI),^19^ segmentation of thalamic subnuclei (version 13)^20^ and hippocampal and amygdala subfields (version 22)^21^ using co‐registered high resolution turbo spin echo coronal T2w imaging (≤0.4 × 0.4 mm in plane). We performed manual quality assessment (QA) for Freesurfer outputs, guided by the QA process used in the Enhancing Neuroimaging Genetics through Meta‐analysis (ENIGMA)‐epilepsy study.^10^ Subjects with more extensive errors, or errors that persisted after repeat processing, were excluded. Finally, subjects with outlier features, as determined by having a feature value greater or less than 1.5 times the interquartile range of the entire cohort, were further visually inspected and manually excluded if there was a significant remaining image quality or processing error.

#### 2.3.2 Imaging features

To select candidate structural imaging features, we referred to group‐level studies reporting structural brain changes in newly diagnosed epilepsy (NDE),^22^ common chronic epilepsy syndromes,^10^ and features reflective of epileptogenic lesions, such as focal cortical dysplasia.^23^

**FIGURE 1:**
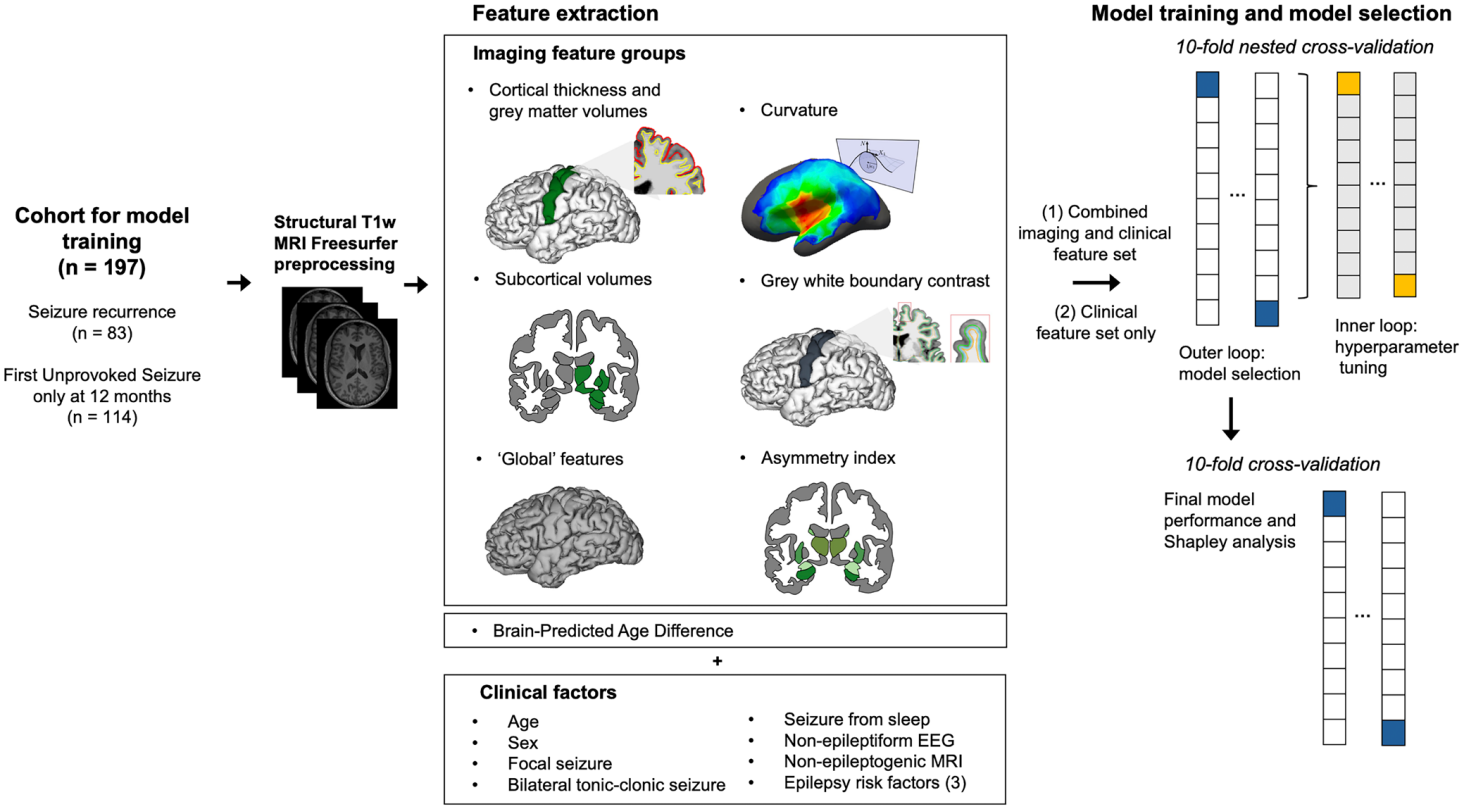
Pipeline for model training and model selection. EEG, electroencephalogram; MRI, magnetic resonance imaging; *n*, number; T1w, T1‐weighted.

In total, 672 FreeSurfer‐derived features were included for model training, grouped into the following seven categories (Figure 1):

1. **Cortical thickness and cortical GM volumes** per cortical region.
2. **Subcortical volumes**—deep GM matter structures from standard *recon‐all* processing, plus finer segmentation of thalamus subnuclei, and amygdala, and hippocampus subfields.
3. **Gyral curvature metrics**—four measures per cortical region to capture folding complexity^24^: (i) mean curvature, (ii) Gaussian curvature, (iii) folding index, and (iv) local gyrification index (LGI; ratio of buried to visible cortex).^19^
4. **Gray‐white boundary contrast**—per cortical region.
5. **Global volumetric and surface metrics**—total and hemispheric intracranial volume, whole‐ or hemisphere‐specific gray/white matter volumes, and surface areas. These measures have been reported as altered in chronic epilepsy.^25^
6. **Asymmetry indices**—calculated for selected bilateral features; altered asymmetry has been observed in neurodevelopmental and neuropsychiatric disorders^26^ relevant to epilepsy.
7. **Brain‐predicted age difference (Brain‐PAD)**—the difference between chronological age and MRI‐derived brain age, estimated using *PyBrainAge* ^27^; increased Brain‐PAD has been reported in established epilepsy.^28^

See Table S3 for complete list of image feature groups, method of calculation, and number of features in each group.

### 2.4 Statistical methods

Study design and prediction model training were performed in accordance with Transparent Reporting of a multivariate prediction model for individual Prognosis Or Diagnosis (TRIPOD) guideline.^29^ All statistical analysis was performed using the *R* programming language in *R* studio (version 4.4.0). Machine learning algorithms were trained and evaluated using the *R* package *mlr3* (version 0.21.0).^30^ Model interpretation was performed using *R* packages *iml* and *explainer* by obtaining Shapley values for each feature in the model for each subject using 500 Monte Carlo permutations to approximate the contribution of each feature to model prediction. Shapley values are an approach that ranks the estimated contribution of each feature to the final classification.^31^ Image harmonization across six scanners was performed using the *NeuroCombat* package (ComBat) within train/test folds to minimize scanner‐related variability.^32^

Two‐tailed model comparisons were performed using the *pROC* package with the DeLong test, a nonparametric method based on U‐statistics.^33^ To determine the optimal classification threshold on the ROC curve, Youden's *J* statistic,^34^ which maximizes the sum of sensitivity and specificity, was applied using the same package.

### 2.5 Model training

Predictive models for seizure recurrence using the combined imaging and clinical data set were trained using four machine learning algorithm classes: Least Absolute Shrinkage and Selection Operator (LASSO), random forest, tree‐based eXtreme Gradient Boosting (XGBoost), and support vector machine (SVM). Given the modest sample size available in this study, we utilized smaller, explainable machine learning algorithms rather than complex neural networks, prioritizing interpretability over the discovery of potentially subtle data‐driven MRI features.

Each algorithm was combined with 1 of 4 filter based feature selection methods: no filter (i.e., all features used), principal components analysis (PCA), AUC filter, and a filter selecting a fixed number of features. This resulted in 16 model architectures (four algorithm classes × 4 feature selection/no feature selection filter methods). Harmonization of imaging predictors was performed within each training fold, with age, sex and the outcome variable (seizure recurrence) assigned as covariates, followed by centring and scaling. A logistic regression clinical feature‐only model was trained using the 10 available categorical clinical features. This approach is the current best published model using clinical features for predicting seizure recurrence.^5^, ^6^, ^35^

### 2.6 Model evaluation and interpretation

A 10‐fold nested cross‐validation (CV) was used for model training and selection. To avoid data leakage and inflated performance estimates, all preprocessing steps, including image harmonization, feature scaling, and feature selection, including dimensionality reduction via PCA, were performed strictly within the training folds of each CV iteration. In the inner loop, a random search strategy was used, with each model architecture evaluated using the same 500 hyperparameter configurations in each inner fold. Following hyperparameter optimization in the inner loop, each model architecture with its best‐performing hyperparameter combination was trained on outer fold train data and tested by obtaining predictions on the corresponding outer fold test data. To obtain the best‐performing model architecture, predictions from the outer 10 test folds were aggregated, and the model with the highest AUC was selected for a final 10‐fold CV. This final 10‐fold CV was then used to obtain the final architecture's average performance and Shapley values. The final clinical feature‐only model also underwent this final CV to enable paired statistical comparison of the AUC.^33^ Feature consistency was assessed by comparing top‐ranked features per fold to the global ranking across all folds, based on mean absolute Shapley values.

See Figure 1 for the analysis pipeline. The hyperparameters and their corresponding search spaces used for hyperparameter optimization can be found in Table S4.

## 3 RESULTS

### 3.1 Cohort

**FIGURE 2:**
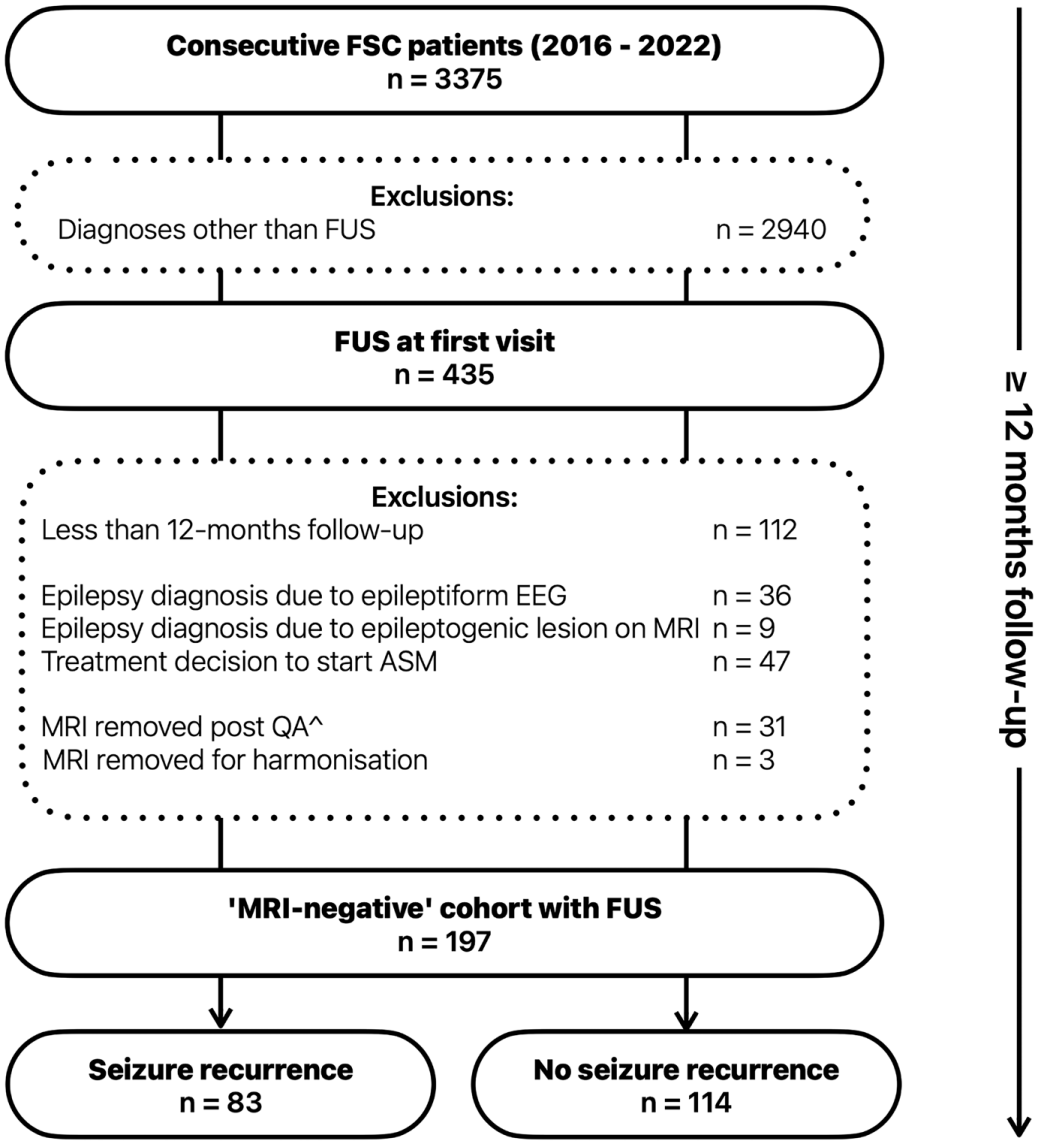
Derivation of cohort for model training. Exclusions are shown in the boxes with dotted borders. ^*n* = 31 MRIs removed (1.5 T *n* = 8, insufficient T1‐weighted and/or coronal T2‐weighted sequences *n* = 6, nonisotropic acquisition *n* = 1, motion degraded *n* = 7, Freesurfer outliers *n* = 9). ASM, anti‐seizure medication; EEG, electroencephalogram; MRI, magnetic resonance imaging; *n*, number; QA, quality assurance.

Cohort selection is shown in Figure 2. From 323 patients with baseline FUS and 12 months or more follow‐up, we excluded patients with epileptiform EEG abnormalities (*n* = 36) and epileptogenic lesion on MRI (*n* = 9) leading to a diagnosis of epilepsy, patients who were commenced on ASM due to clinical judgment (*n* = 47) and patients with MRI images of poor quality or insufficient for harmonization (*n* = 34). The final cohort of 197 patients comprised 83 who had seizure recurrence; mean age 43.5 (18.9) years, 57% male, and 114 who had FUS only (*n* = 114); mean age 39.1 years, standard deviation (SD) 18.5 years at 12‐month follow‐up. Demographic and clinical features of the cohort are shown in Table 1.

**TABLE 1:** Cohort for algorithm training.

	FUS, with seizure recurrence within 12 months	FUS only, no seizure recurrence within 12 months
Number of patients	83	114
Age, mean (SD) years	39.1 (18.5)	43.5 (18.9)
Sex, *n* (%)		
Male	47 (57%)	65 (57%)
Female	36 (43%)	49 (43%)
Seizure with focal semiology (includes FIC and FBTC), *n* (%)	19 (23%)	37 (32%)
Seizure with tonic–clonic semiology (includes BTC and FBTC), *n* (%)	82 (99%)	104 (91%)
Seizure from sleep, *n* (%)	35 (42%)	27 (24%)
EEG, *n* (%)		
Normal	69 (83%)	102 (89%)
Non‐epileptiform EEG abnormality	14 (17%)	12 (11%)
Epileptiform EEG abnormality	0 (0%)	0 (0%)
MRI, *n* (%)		
Normal	51 (61%)	73 (64%)
Non‐epileptogenic MRI abnormality	32 (39%)	41 (36%)
Epileptogenic MRI abnormality	0 (0%)	0 (0%)
FLAIR imaging for processing with T1w imaging^a^	60 (72%)	89 (78%)
Epilepsy risk factors, *n* (%)		
No epilepsy risk factors identified	57 (69%)	77 (68%)
First‐degree relative with epilepsy	10 (12%)	9 (8%)
Developmental encephalopathy	3 (4%)	4 (4%)
Neurodegenerative disorder	7 (8%)	5 (4%)

Abbreviations: BTC, bilateral tonic–clonic seizure unknown whether focal or generalized onset; EEG, electroencephalography; FBTC, focal to bilateral tonic–clonic seizure; FIC, focal impaired consciousness seizure; FLAIR, fluid‐attenuated inversion recovery; FUS, first unprovoked seizure; MRI, magnetic resonance imaging; *n*, number; SD, standard deviation; T1w, T1‐weighted MRI.

^a^ FLAIR availability was not associated with differences in model performance (accuracy with FLAIR: 0.60 ± 0.23; without FLAIR: 0.62 ± 0.12; Wilcoxon V = 28, *p* = 1).

### 3.2 Model architecture selection from nested cross‐validation

Model performance from nested CV showed that the best‐performing model was a radial‐basis‐kernel SVM (*cost* 17.98, *gamma* 0.0001428, no feature selection filter), incorporating both imaging and clinical features, yielding an AUC 0.65; 95% confidence interval (CI) 0.59–0.70. The clinical feature‐only logistic regression model achieved an AUC 0.57 (95% CI 0.49–0.66).

**FIGURE 3:**
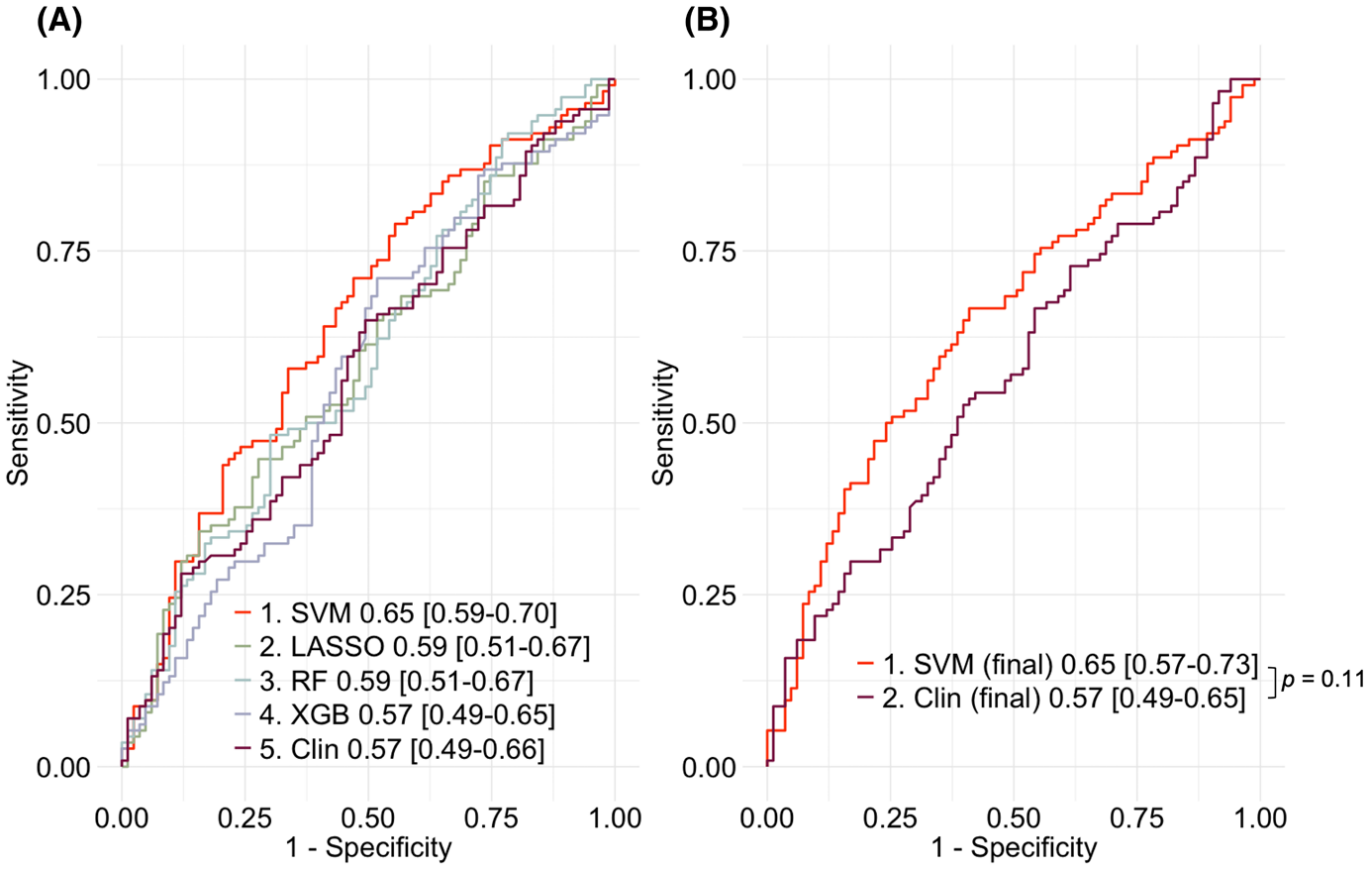
(A) Nested cross‐validation model performance for model architecture selection, showing the highest AUC [95% confidence interval] of each model algorithm class; models 1–4 were trained on a combination of imaging and clinical features, and model 5 is the clinical feature‐only logistic regression model, selecting the SVM as the highest performing model. (B) Final cross‐validation model performance of the selected SVM combined imaging and clinical feature model versus clinical feature‐only model. AUC, area under receiver operating characteristic curve; Clin, clinical feature‐only model; final, final 10‐fold cross‐validation; LASSO; Least Absolute Shrinkage and Selection Operator; RF, random forest; SVM, support vector machine; XGB, eXtreme Gradient Boosting.

The highest AUC for the remainder of each machine learning algorithm type was LASSO with PCA variance filter 0.59 (CI 0.51–0.67), random forest with feature number filter 0.59 (CI 0.51–0.67), and XGBoost with AUC filter 0.57 (CI 0.49–0.65). See Figure 3A for nested CV AUC performance. A summary of the combined T1w‐clinical models and clinical factor‐only logistic regression model is provided in Tables S5 and S6, respectively. Performance for clinical‐only machine learning models and T1w–only models is provided in Figure S1.

### 3.3 Final model performance

The final CV performance for the SVM combined T1w‐clinical model yielded a higher AUC 0.65 (95% CI 0.57–0.73) versus the final clinical feature‐only model AUC 0.57 (CI 0.49–0.65). The difference in the AUC of these two models was not statistically significant (DeLong test: *p* = 0.11; 95% CI for the difference in AUC: −0.019 to 0.173; see Figure 3B). Using the Youden's *J* statistic for the optimal sensitivity‐specificity threshold, the SVM model demonstrated a positive predictive value (PPV) of 0.53 and negative predictive value (NPV) of 0.74, compared with the clinical feature‐only model's PPV of 0.46 and NPV of 0.71. A calibration plot of the SVM model can be found in Figure S2.

### 3.4 Influential features of the best‐performing model

In the best‐performing model, the most influential feature groups, based on global feature importance ranking across all folds (highest mean absolute Shapley values), in descending order, were clinical features, followed by cortical curvature, asymmetry indices, cortical GM volumes, subcortical volumes, gray‐white boundary contrast, and the hemispheric/whole‐brain “global” features.

**FIGURE 4:**
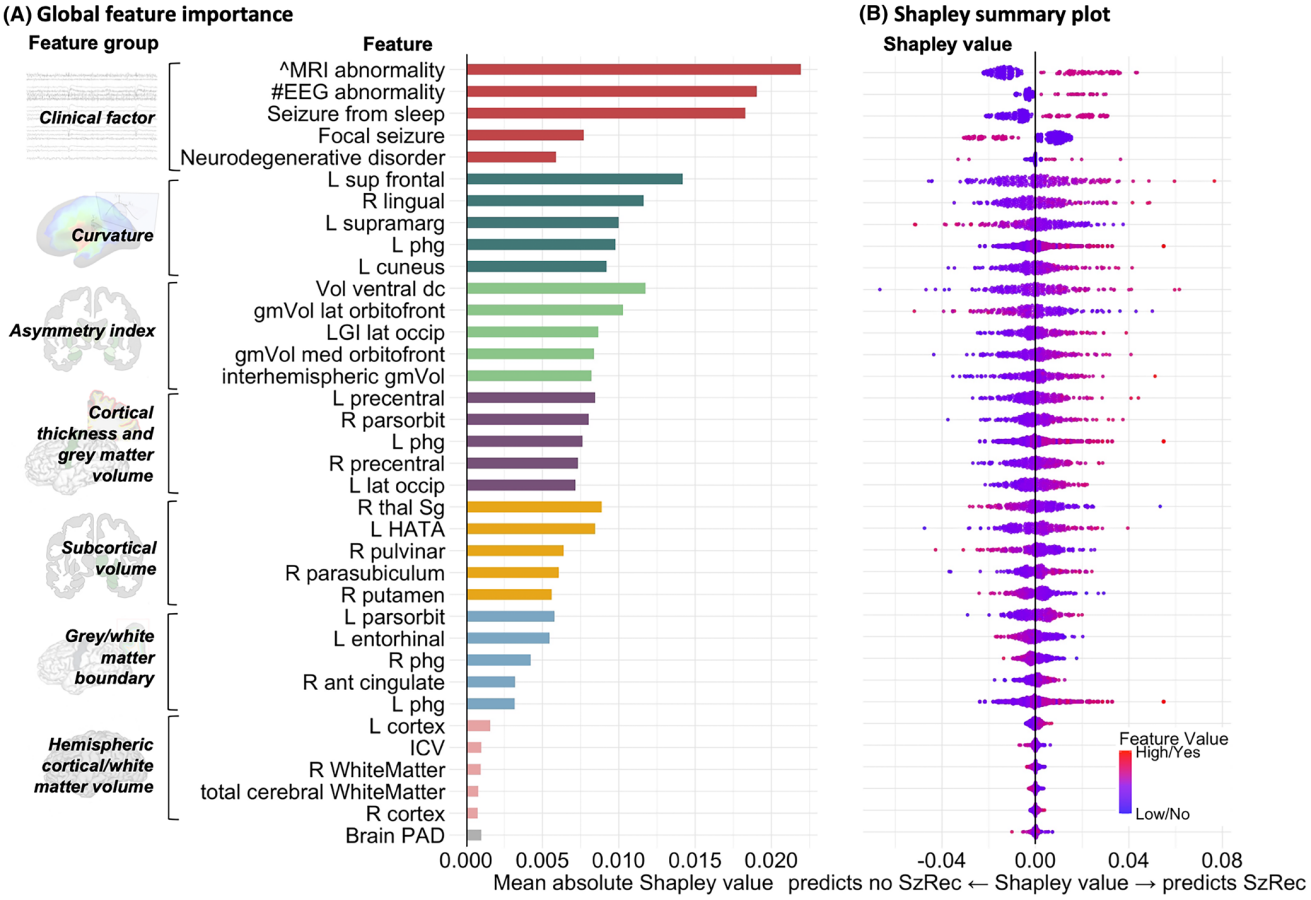
Feature importance and contribution to seizure recurrence prediction. (A) Global feature importance of the top 5 features in each feature group, ordered from highest to lowest ranked feature groups and features, based on absolute mean Shapley values for each feature across all subjects; (B) Shapley summary plot showing the distribution with the Shapley value for each feature, negative Shapley values predict no seizure recurrence and positive values predict recurrence. Color is used to display the scaled original value of the feature (red – higher value, blue – lower value). The width of each violin reflects the density of Shapley values. For example, in figure 4A, within the curvature feature group, the left superior frontal gyrus feature demonstrates the highest feature importance of all imaging features. In the corresponding Shapley summary plot (4B), higher values of this feature (red dots, reflecting increased complexity of cortical folding) are associated with positive Shapley values, indicating an increased contribution to seizure recurrence prediction. Brain‐PAD, brain‐predicted age difference; dc, diencephalon; gmVol, gray matter volume; HATA, hippocampal‐amygdala transition area; ICV, intracranial brain volume; L, left; lat, lateral; LGI, local gyrification index; med, medial; occip, occipital; orbitofront, orbitofrontal; parsorbit, parsorbitalis; phg, parahippocampal gyrus; R, right; sup, superior; supramarg, supramarginal; SzRec, seizure recurrence; thal Sg, suprageniculate nucleus of thalamus; Vol, volume; ^non‐epileptogenic MRI abnormality, #non‐epileptiform EEG abnormality.

Binary clinical variables of nonepileptogenic MRI, nonepileptiform EEG abnormalities, and seizures from sleep emerged as the highest ranked features. Of the top‐ranking imaging features, curvature features that were most predictive included folding index of the left superior frontal gyrus, supramarginal gyrus, and parahippocampal gyrus. Asymmetry indices with high feature importance were contributed by subcortical and cortical GM, including the ventral diencephalon (includes hypothalamus), orbitofrontal cortex, inter‐hemispheric GM, and curvature of the lateral occipital cortex. Important cortical thickness and GM volumes included cortical thickness of right pars orbitalis, bilateral precentral gyri, and left lateral occipital gyrus. Important subcortical regions included the thalamic subnuclei (left suprageniculate nucleus and right pulvinar) and hippocampal subfields (left hippocampal‐amygdala transition area, right parasubiculum), and right putamen volume. Overall contribution of Brain‐PAD to the model was minimal. See Figure 4 for a summary of (A) feature importance and (B) Shapley values.

Across the 10 folds, a median of 11 (range 8–13) of the top 20 globally ranked features per fold overlapped, showing reasonable stability of feature importance. Post hoc group‐level testing revealed significant differences in 9 of the top 20 globally ranked imaging features after multiple comparison correction (Figure S3).

**FIGURE 5:**
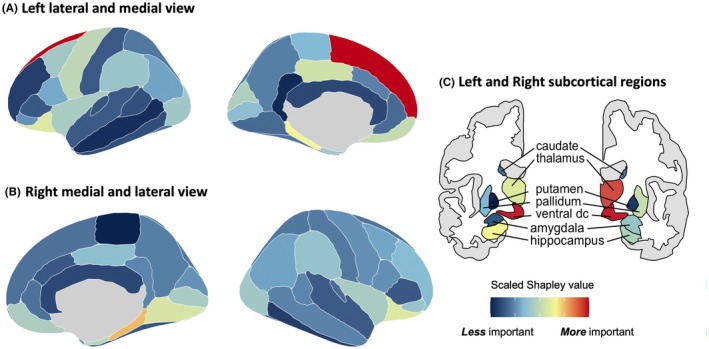
Feature importance of cortical and subcortical regions. Colors represent scaled absolute Shapley values derived from the feature with the maximum absolute Shapley value within each atlas region. Panels show (A, B) cortical and (C) subcortical regions. Higher scaled Shapley values (shown in red) indicate regions with greater overall contribution to model predictions, while lower values (shown in blue) indicate relatively lower importance. Dc, diencephalon.

Noteworthy regions as highlighted on surface cortical and subcortical atlases (Figure 5) were the frontal (including orbitofrontal, superior frontal gyri, precentral gyri), parietal (pars orbitalis, supramarginal gyrus, paracentral gyrus), and occipital lobes (lateral occipital, lingual gyrus), with more left hemispheric features compared with right. Key subcortical regions included limbic structures, such as the ventral diencephalon, left hippocampus, and right thalamus.

## 4 DISCUSSION

The aim of this study was to derive a prediction model for 12‐month seizure recurrence after FUS using structural T1w MRI, and from this identify potential brain biomarkers which may improve prediction of seizure recurrence. Using a well‐characterized multicenter cohort of patients with a FUS, who have normal or nondiagnostic EEG and MRI, we trained a machine learning classifier on a combined dataset of structural MRI features extracted from automated processing and clinical features. Overall, the results show that quantitative metrics from anatomical imaging contribute additional predictive value for seizure recurrence. Using a robust nested CV approach, we found that a SVM was the best‐performing architecture and demonstrated a final AUC of 0.65 (95% CI 0.57–0.73). This increment in predictive performance is promising compared with a logistic regression clinical feature‐only model, which yielded an AUC of 0.57 (95% CI 0.49–0.65), even though direct comparison between these models did not achieve statistical significance (*p* = 0.11).

Quantitative image analysis in this study provides evidence that adults have structural brain changes at the time of FUS which have prognostic relevance to seizure recurrence. The features most predictive for seizure recurrence belonged to feature groups of curvature, asymmetry, and cortical GM volume. Abnormalities in cortical curvature are well‐recognized in epilepsy, ranging from subtle abnormalities harboring focal cortical dysplasia,^36^ to extensive malformations of cortical development, such as polymicrogyria. In our work, inter‐hemispheric asymmetry contributed by subcortical and cortical GM volumes, and LGI (a metric of cortical curvature) emerged as important biomarkers for seizure recurrence. Within‐individual asymmetry allows a given subject to be their own “baseline,” facilitating detection of atypical patterns regardless of side.^37^ In studies of temporal lobe epilepsy (TLE), asymmetry patterns appear less correlated with duration of epilepsy and age of onset and may reflect different pathological processes, such as an initial regional insult to the limbic system.^38^, ^39^ Our finding of asymmetry indices contributed by individual alterations in GM and gyral curvature being predictive suggests that subtle but diffuse regional structural brain alterations may predate the clinical manifestation of epileptogenesis (i.e., the time of first seizure). In the context of seizure recurrence risk, hemispheric asymmetries may reflect a variety of factors, such as preexisting developmental differences, age‐related changes, or acquired causes.^26^ Structural alterations from a single seizure are considered possible but less likely.^40^ Our findings are consistent with evidence that altered asymmetry has clinical relevance in long‐standing epilepsy^41^ and other neurodevelopmental and neuropsychiatric conditions,^26^ and suggest that atypical hemispheric patterns may reflect individual vulnerability to seizure recurrence.

Regarding GM volume and cortical thickness changes, GM alterations may have possible contributions from epilepsy duration, ASM use and seizure frequency^42^ in long‐standing epilepsy, though these factors are less relevant to ASM‐naïve patients experiencing a single seizure. However, cortical GM thinning has also been shown to occur independently of these factors, with areas of accelerated cortical thinning identified in patients with new onset focal epilepsy within 5 years of initial seizure,^43^ consistent with some of the patterns seen in our cohort.

We observed that structural features of high importance align with key functional brain networks. Specifically, structural features that comprise the fronto‐parietal cortex, limbic system, language‐related areas, and visuospatial processing systems were most influential in the final model. These regions correspond to the location of structural alterations observed in long‐standing focal and generalized epilepsy.^44^ First, studies in NDE and idiopathic generalized epilepsy (IGE) have found reduced connectivity in the fronto‐parietal attention networks, particularly in the frontal regions compared with controls.^44^, ^45^ Second, we found a predominance of left compared with right hemispheric features, particularly those overlapping with classical language systems, such as the superior frontal gyrus, pars orbitalis, supramarginal gyrus, and insula.^46^ Asymmetrical leftward intra‐hemispheric connectivity is thought to be closely related to higher order language function,^47^ and our findings suggests that regions that subserve language, a network vulnerable in TLE,^48^, ^49^ contribute to prediction. Thirdly, parieto‐occipital regions such as the lateral occipital cortex, lingual gyrus, cuneus, are related to visuospatial function, shown to be variably involved in TLE^50^ and IGE,^51^ and are particularly vulnerable to developmental and acquired insults (e.g., hypoglycemia, hypoxia) which are known to contribute to epileptogenesis.^52^ Last, subcortical regions that also influenced the model's predictions, including ventral diencephalon, parahippocampal gyrus, anterior cingulate, entorhinal, anterior thalamic nuclei, and hippocampal regions, have a well‐described role in seizure propagation and epileptogenesis in humans and animal models.^53^, ^54^

Clinical factors remained the most predictive feature group in the combined model. Nonepileptogenic MRI, nonepileptiform EEG abnormalities, and index seizure from sleep were particularly relevant for estimating seizure recurrence risk. In our cohort, index seizure without focal onset (i.e., BTC, unknown if focal or generalized) was a stronger predictor than a focal seizure (FIC and FBTC), differing from some prior studies suggesting a higher risk of recurrence of focal seizures compared with generalized seizures, especially in those with prior neurological injury.^1^ Our findings may reflect inclusion of an MRI‐negative only cohort, where patients with clear focal structural pathology were excluded, and the greater diagnostic certainty of a witnessed BTC compared with a focal seizure. Notably, of the small number of patients with FIC in our cohort, the majority surprisingly did not have seizure recurrence. Consequently, the model may have favored BTC as a predictor of recurrence because these cases were more definitively identified.

The presence of a nonepileptogenic MRI abnormality had the highest feature importance among all clinical and imaging features, suggesting that radiologist‐reported abnormalities carry prognostic information when considered collectively, albeit not in isolation. The most common reported findings included nonspecific T2w FLAIR white matter hyperintensities (either diffuse or focal and moderate–severe), global cerebral atrophy, and hippocampal asymmetries in signal or volume without overt sclerosis. Given the heterogeneous findings on a small subset of patients, and that all these findings were expressed as a single binary factor, the extent of increased risk from each finding is difficult to determine. Clarifying the predictive value of specific equivocal or incidental MRI findings will require larger cohorts with high‐quality imaging. Notably, as the most frequent abnormalities involved nonspecific T2w FLAIR white matter hyperintensities, hippocampal and amygdala signal asymmetries without overt sclerosis, our findings support the potential utility of incorporating quantitative T2w FLAIR–derived features in future seizure recurrence prediction models.

Estimated brain age, using Brain‐PAD index, has been shown to be slightly increased in NDE, by an average of 1 year, and is more markedly increased in drug‐refractory focal epilepsy.^28^, ^55^ Nevertheless, we found it had relatively limited predictive value at the time of FUS compared with the other features we considered. There is some evidence that earlier age of epilepsy onset, frequent seizures, longer duration of epilepsy, and ASM use may increase Brain‐PAD.^28^, ^55^ In our ASM‐naïve cohort, we postulate that limited seizure frequency and shorter epilepsy duration of 12 months may explain why Brain‐PAD was not a strong predictor.

Key strengths of our study include the careful assessment of EEG and MRI findings before patient inclusion, while still including those with equivocal and nondiagnostic results commonly encountered in clinical practice. Excluding individuals who already have diagnostic findings of epilepsy on these tests, and ensuring all participants were ASM‐naïve, are important points of difference between our study design and historical first seizure studies.^56^, ^57^ This design addresses the cohort with the greatest clinical uncertainty about seizure recurrence. The resulting model demonstrated a PPV of 0.53 and NPV of 0.74, suggesting greater utility for identifying individuals at lower risk of seizure recurrence than for confidently predicting recurrence. Clinically, this may support counseling patients who are unlikely to recur in the short term, with potential to avoid overdiagnosis of epilepsy and unnecessary ASM use. Importantly, calibration shows good agreement between predicted and observed risk, particularly around clinically relevant thresholds such as the 60% recurrence risk used in the ILAE definition of epilepsy,^2^ supporting interpretability of model outputs despite modest discrimination. However, the modest PPV limits its utility in settings where high certainty of recurrence risk is required, such as decisions related to high‐risk occupations or driving, highlighting the need for better prognostic models and biomarkers in this context.

In the relatively new era of machine learning‐guided outcome prediction in epilepsy, there are so far only a few studies that extend beyond clinical factors and used quantitative biomarkers to predict seizure recurrence after a FUS. A recent model using 1.5 T MRI with hippocampal imaging yielded an AUC of 0.92.^58^ However, the study included individuals with epileptogenic lesions in their cohort, including visually apparent hippocampal sclerosis and subcortical band heterotopia, findings already known to be predictive of seizure recurrence. Other cohorts have shown encouraging results using EEG in combination with advanced imaging techniques. A study applying machine learning to quantitative features on routine clinical EEG yielded a nested cross‐validated AUC of 0.63 for predicting 12‐month seizure recurrence after FUS in patients without visually apparent epileptiform EEG abnormalities,^59^ similar to the performance observed in our internally validated model. In a smaller cohort of 24 patients, another study combined functional MRI and quantitative EEG features to obtain an AUC of 0.75 for seizure recurrence on a hold‐out test cohort of 5 patients.^60^ Our findings, alongside prior research, highlight that developing prediction tools in epilepsy requires close clinician and imaging collaboration, well‐characterized cohorts, high‐quality MRI, and robust unbiased machine learning methods that are likely to provide performance estimates reflective of real‐world utility.

### 4.1 Limitations

First, we have defined our prediction model for 12‐month seizure recurrence, noting that previous landmark studies have shown that a substantial proportion of individuals who experience recurrence do so within the first year after a FUS (cumulative risk approximately 37% at 1 year, 51% by 8 years),^61^ therefore, a 12‐month timeframe is clinically relevant. Training additional prediction tools on 2‐year or 5‐year data would have clinical utility, though it brings the challenges of obtaining unbiased long‐term follow‐up data.

Secondly, we adopted a retrospective study design, using high quality but heterogenous clinically acquired imaging, reflecting the “real world” context. We applied ComBat harmonization for six scanners, implemented within training folds to avoid data leakage and inflation of test performance.^62^ While harmonization improves generalizability of results, some residual scanner‐related differences may remain, potentially introducing additional variability into the data.

Third, the total number of subjects can still be considered small relative to the number of predictors, creating some risk of overfitting despite the use of feature reduction approaches. While some variability in Shapley feature rankings is expected in high‐dimensional datasets, we demonstrated that a consistent set of clinical, cortical folding, and asymmetry features contributed to prediction, with many top imaging features showing significant group differences on post hoc testing. Thus, while biological interpretations should be made cautiously, these features reflect biological plausibility and guide future refinement of imaging biomarkers for seizure recurrence prediction.

Finally, validating this model against an independent external cohort would be essential for clinical translation, but given the current performance is still insufficient for deployment in the clinic, we suggest that further model development to improve predictive performance should be conducted first. Future model refinement is likely to require even larger cohorts, as evidenced by successful clinical prediction models developed in other disorders, such as dementia.^63^

### 4.2 Future directions

Despite modest overall performance of the best models in this study, the increment obtained by adding structural imaging features is a promising result. Our findings suggest that further improvements in performance are likely to be achieved by harnessing additional imaging and nonimaging modalities. For example, prior work has shown that qualitative EEG features,^59^ functional MRI^60^ and genetic markers, such as epilepsy polygenic risk scores,^64^ demonstrate some prognostic value following a FUS. Thus, multimodal and complementary sources of information, in larger and prospectively acquired cohorts, represent a crucial step toward improving the clinical utility of seizure recurrence prediction models.

### 4.3 Conclusions

Quantitative imaging features from T1‐weighted structural MRI can add additional information to prediction models of seizure recurrence after a FUS, though clinical features remain essential. GM cortical and subcortical hemispheric asymmetries and changes to cortical curvature, especially in the fronto‐parietal and limbic regions, were most important for prediction in this study, supporting the role of these subtle brain changes as prognostic biomarkers for seizure recurrence risk after FUS.

## AUTHOR CONTRIBUTIONS

S.O. was involved in ethics approval, data acquisition, experimental design, analysis, interpretation of the data and drafting the manuscript. C.T. was involved in conceptualization of study, experimental design and interpretation of the data. H.R.P. was involved in data acquisition and interpretation of the data. P.W.C was involved in ethics approval and data acquisition. M.S. was involved in ethics approval and data acquisition. J.H. was involved in experimental design and interpretation of the data. G.D.J was involved in conceptualization of study and final approval of the manuscript to be published. D.N.V was involved in conceptualization of the study, ethics approval, data acquisition, experimental design, and final approval of the manuscript to be published. All authors were involved in critically revising the manuscript.

## FUNDING INFORMATION

S.O. is supported by a National Health and Medical Research Council (NHMRC) postgraduate scholarship (project ID 2022072) and the Australian New Zealand Association of Neurologists (ANZAN) Education and Research Fund. D.N.V. and C.T. are supported by an NHMRC project grant (APP1157145). C.T., H.R.P., J.H., G.D.J. and D.N.V. are supported by the Australian Epilepsy Project, funded by the Australian Government Medical Research Future Fund, Grant/Award Number: MRFF75908 and RFRHPSI000008; Victorian‐led Frontier Health and Medical Research Program.

## CONFLICT OF INTEREST STATEMENT

The authors declare that they have no conflict of interest. We confirm that we have read the Journal’s position on issues involved in ethical publication and affirm that this report is consistent with those guidelines.

## ETHICAL APPROVAL

Approval was granted by local Human Research Ethics Committees of Austin Health – HREC/57333/Austin‐2019, Eastern Health – S23‐019‐57 333, and Northern Health – SSA/57333/NH‐2023.

## PATIENT CONSENT STATEMENT

Patient data from hospital medical records was used in this study under a waiver of consent, as approved by local Human Research Ethics Committees. In cases where further patient contact was required, additional written informed consent was obtained.

## Supporting information


Data S1:

## Data Availability

The data that support the findings of this study are available on request from the corresponding author. The data are not publicly available due to privacy or ethical restrictions.
