# Supplementary material for: Structural brain imaging biomarkers for predicting seizure recurrence after a first unprovoked seizure
Source: Epilepsia Open. 2026 Feb 17;11(2):538–52. doi: 10.1002/epi4.70236 (PMC13052194; doi:10.1002/epi4.70236)
Supplement: Supplementary file 1 — Data S1: [file EPI4-11-538-s001.docx]

**Supporting information tables:**

Supporting information table S1: (a) Types of non-epileptogenic MRI abnormalities reported in cohort,

(b) Types of non-epileptiform EEG abnormalities reported in cohort

Supporting information table S2: MRI scanner site, type and acquisition parameters

Supporting information table S3: Freesurfer-derived imaging features included in model training

Supporting information table S4: (a) Hyperparameter ranges for each model algorithm type,

(b) filter based feature selection method

Supporting information table S5: Summary of nested cross validation model performance with the best model performance of each model algorithm type/feature selection filter

Supporting information table S6: Summary of final clinical factor-only logistic regression model

**Supporting information figures:**

Supporting information figure S1: Nested cross-validated performance of imaging-only and clinical-only models

Supporting information figure S2: Calibration plot of the best performing model

Supporting information figure S3: Group-level differences in imaging features with highest global feature importance

**Supporting information** **table S1a: Non-epileptogenic MRI abnormality reported in cohort***

| **Non-epileptogenic MRI abnormality** | **Seizure recurrence within 12 months, n (% of 83 cases)** | **No seizure recurrence within 12 months), (% of 114 controls)** |
| --- | --- | --- |
| White matter hyperintensities | 16 (19%) | 21 (18%) |
| Focal white matter hyperintensities | 5 (6%) | 10 (9%) |
| Global atrophy | 4 (5%) | 11 (10%) |
| Focal atrophy | 0 (0%) | 1 (1%) |
| HIMAL | 3 (4%) | 3 (3%) |
| Hippocampal abnormality (excluding HS)** | 2 (2%) | 4 (4%) |
| Cysts: arachnoid, pineal, Rathke cleft, ependymal | 3 (4%) | 3 (3%) |
| Small areas of cortical or subcortical ischaemia | 0 (0%) | 4 (4%) |
| Meningocele | 1 (1%) | 2 (2%) |
| Arteriovenous malformation | 2 (2%) | 1 (1%) |
| Focal SWI abnormality | 3 (4%) | 0 (0%) |
| Encephalocele (< 5 mm, no cortical gliosis) | 1 (1%) | 1 (1%) |
| Non-specific gliosis | 2 (2%) | 0 (0%) |
| Excessive perivascular spaces | 1 (1%) | 1 (1%) |
| Ventriculomegaly | 1 (1%) | 1 (1%) |
| Amygdala abnormality (size and/or signal) | 1 (1%) | 1 (1%) |
| Focal mass# | 1 (1%) | 2 (2%) |
| Other subtle cortical abnormality^ | 1 (1%) | 2 (2%) |

*more than one abnormality in some individuals

**atrophy, signal change, dysplasia, asymmetry, hippocampal cysts

#E.g. subependymal nodule, haemangioma, small meningioma without oedema/gliosis

^E.g. focal cortical thickening or asymmetrical gyral pattern without other definite features of malformations of cortical development

**Abbreviations**: MRI – magnetic resonance imaging, FUS – first unprovoked seizure, n – number, HIMAL – hippocampal inversion and malrotation, HS – hippocampal sclerosis, SWI – susceptibility weighted imaging

**Supporting information** **table S1b: Non-epileptiform EEG reported in the cohort**

| **Non-epileptiform EEG abnormality** | **Seizure recurrence within 12 months, n (% of 83 cases)** | **No seizure recurrence within 12 months, n (% of 114 controls)** |
| --- | --- | --- |
| Focal slowing | 4 (5%) | 5 (4%) |
| Generalised slowing | 6 (7%) | 2 (2%) |
| Photoparoxysmal response confined to the parieto-occipital regions | 1 (1%) | 2 (2%) |
| Other | 3 (4%) | 3 (3%) |

**Abbreviations**: EEG – electroencephalogram, n – number

**Supporting information** **table S2: MRI scanner site, type and acquisition parameters**

| **Site** | **Scanner** | **Sequence** | **SzRec (n)** | **FUS-only (n)** | **TR (ms)** | **TE (ms)** | **TI (ms)** | **FA (degrees)** | **Voxel (mm)** | **Dimensions (mm)** |
| --- | --- | --- | --- | --- | --- | --- | --- | --- | --- | --- |
| AH | Skyra | 3D T1w MPRAGE | 30 | 39 | 2000 – 2300 | 1.82 – 2.98 | 900 | 7 – 9 | 0.8 x 0.8 x 0.8 | 256 x 256 x 256 |
| AH | Prisma Fit | 3D T1w MPRAGE | 11 | 13 | 1900 | 2.19 | 850 - 990 | 8 | 0.9 x 0.9 x 0.9 | 192 x 256 x 256 |
| EH | Magnetom Vida | 3D T1w MPRAGE | 2 | 4 | 1900 – 2200 | 2.99 – 3.06 | 950 | 8 | 0.9 x 0.9 x 0.9 | 208 x 256 x 256 |
| EH | Skyra Fit | 3D T1w MPRAGE | 4 | 5 | 2300 | 2.32 | 920 | 8 | 0.9 x 0.9 x 0.9 | 256 x 208 x 256 |
| EH | Ingenia | 3D T1w TFE | 19 | 34 | 7.0 – 11.6 | 3.76 – 4.97 | NA | 8 | 0.9 x 0.7 x 0.7 | 240 x 384 x 384 |
| NH | Discovery | 3D T1w SPGR | 17 | 19 | 8.7 | 2.31 – 3.45 | 450 | 8 – 12 | 0.9 x 1.0 x 0.9 | 256 x 196 x 256 |

**Abbreviations**: MRI – magnetic resonance imaging, AH – Austin Health, EH – Eastern Health, NH – Northern Health, ms – milliseconds, seconds (s), TR – repetition time, TE – echo time, TI – inversion time, FA – flip angle, MPRAGE - Magnetization Prepared Rapid Gradient Echo Imaging, n – number of patients, TFE – turbo fast echo, SPGR – spoiled gradient echo, FUS-only– first unprovoked seizure only at 12 months, SzRec – seizure recurrence at 12 months

**Supporting information** **table S3: Freesurfer-derived imaging features included in model training**

| Feature group | Total | Right | Left | Midline structures |
| --- | --- | --- | --- | --- |
| Cortical grey matter* |  |  |  |  |
| Cortical grey matter thickness | 70 | 35 | 35 |  |
| Cortical grey matter volume | 70 | 35 | 35 |  |
| Subcortical volumetry | 116 | 58 | 58 |  |
| Subcortical basal ganglia | 4 | 2 | 2 |  |
| Corpus callosum | 5 |  |  | 5 |
| Curvature |  |  |  |  |
| Folding index | 70 | 35 | 35 |  |
| Mean gaussian | 68 | 34 | 34 |  |
| LGI | 68 | 34 | 34 |  |
| Mean curvature | 6 | 3 | 3 |  |
| Whole brain/white matter |  |  |  |  |
| ICV | 1 |  |  |  |
| Subcortical grey matter volume | 1 |  |  |  |
| Total grey matter volume | 1 |  |  |  |
| Total surface area | 2 | 1 | 1 |  |
| Cortex volume | 2 | 1 | 1 |  |
| Cerebral white matter volume | 3 | 1 | 1 |  |
| Cortical white matter surface area | 2 | 1 | 1 |  |
| Grey-white matter boundary contrast^#^ | 68 | 34 | 34 |  |
| Asymmetry indices** | 114 |  |  |  |
| Brain-PAD | 1 |  |  |  |
| total | 672 |  |  |  |

*Cortical thickness is calculated as the closest distance from the grey-white boundary to the grey-CSF boundary at each vertex on the tessellated surface. Grey matter (GM) volumes are calculated from the sum of the product of the area of the vertex on the pial white matter (WM) surface by the cortical thickness at that vertex

#Grey-white matter boundary contrast: calculated from the vertex-by-vertex percent contrast between GM and WM, where contrast = [100*(WM – GM)] /[0.5*(WM + GM)]. WM is sampled 1mm below the grey-white surface. GM is sampled 30% of the thickness into the cortex.

**Asymmetry index, calculated by dividing the Left (L) – Right (R) feature value, by the L + R feature value, (L – R)/(L + R).

**Abbreviations**: LGI – local gyrification index, ICV – intracranial brain volume, Brain-PAD – brain predicted age difference

**Supporting information** **table S4: (a) Hyperparameter ranges for each model algorithm type and (b) filter based feature selection method**

(a) Model algorithms with the following hyperparameter ranges were used with a random search strategy with the same 500 evaluations per 10 inner folds:

| **Machine learning model algorithm type** | **Hyperparameter and range** |
| --- | --- |
| Radial SVM | cost ∈ 2^-4^ to 2^6^, gamma ∈ 2^-15^ to 2^-6^ |
| Random Forest | number of trees ∈ 400 to 700, mtry ∈ 15 to 35 |
| LASSO | lambda ∈ 2^-7^ to 2^-3^ |
| XGBoost | nrounds ∈ 200 to 600, eta ∈ 0.001 to 0.4, gamma ∈ 0 to 10, max depth ∈ 5 to 12, minimum child weight ∈1 to 10, alpha = 1 |

(b) Each model algorithm type was combined with each of the following filter-based feature selection methods resulting in 16 (4 x 4) model architectures:

| **Filter-based feature selection method** | **Description** | **Hyperparameter and range** |
| --- | --- | --- |
| Feature importance number filter | Applies an importance filter to select number of features based on importance ranking by ‘mode’ (the most common importance ranking across resamples) | Number of features as a hyperparameter ∈ 100 to 690 |
| PCA filter | Applies a PCA to all the data, and then selects features based on variance fraction | PCA variance fraction ∈ 0.5 to 0.9 |
| AUC filter | Applies a filter selecting individual feature discrimination between classes based on AUC. | Absolute value of the difference between an AUC of 0.5 ∈ 0.1 to 0.3 |
| No filter selection method | NA | NA |

**Abbreviations**: SVM – support vector machine, RF – random forest, LASSO - Least Absolute Shrinkage and Selection Operator, XGB – eXtreme Gradient Boosting, AUC – area under the receiver operating characteristic curve, PCA – principle components analysis, NA – not applicable

**Supporting information** **table S5: Summary of nested cross validation model performance with the best model performance of each model algorithm type/feature selection filter**

Positive predictive value (PPV), negative predictive value (NPV), sensitivity and specificity were calculated by obtaining the optimal classification threshold on the ROC curve using Youden’s J statistic.

| **Model algorithm class/feature selection filter** | **AUC (95% CI)** | **PPV** | **NPV** | **Sensitivity** | **Specificity** |
| --- | --- | --- | --- | --- | --- |
| Radial SVM/no filter | 0.65 (0.59-0.70) | 0.53 | 0.70 | 0.66 | 0.58 |
| Random Forest/no filter | 0.59 (0.51-0.67) | 0.50 | 0.69 | 0.70 | 0.48 |
| LASSO/PCA filter | 0.59 (0.51-0.67) | 0.48 | 0.75 | 0.84 | 0.34 |
| XGBoost/PCA filter | 0.57 (0.49-0.65) | 0.55 | 0.65 | 0.71 | 0.48 |
| Clinical model | 0.57 (0.49-0.66) | 0.47 | 0.76 | 0.88 | 0.28 |

**Abbreviations**: SVM – support vector machine, RF – random forest, LASSO - Least Absolute Shrinkage and Selection Operator, XGB – eXtreme Gradient Boosting, Clin – clinical feature-only model, AUC – area under receiver operating characteristic curve, PCA – principle components analysis, CI – 95% confidence interval

**Supporting information** **table S6: Summary of final clinical feature-only logistic regression model**

| **Clinical predictor** | **Odd Ratio (95% CI)** | ***p*-value** |
| --- | --- | --- |
| Tonic clonic seizure | 2.78 (0.40 – 56.35) | 0.37 |
| Seizure from sleep | 2.85 (1.38 – 6.07) | 0.005 |
| First degree family history of epilepsy | 1.41 (0.25 – 7.15) | 0.68 |
| Non-epileptiform EEG abnormality | 1.96 (0.75 – 5.22) | 0.17 |
| Focal onset seizure | 1.15 (0.52 – 2.51) | 0.73 |
| Non-epileptogenic MRI finding | 1.59 (0.80 – 3.21) | 0.18 |
| Sex | 0.95 (0.49 – 1.82) | 0.87 |
| Neurodegenerative disorder | 0.73 (0.18 – 2.68) | 0.65 |
| Age of first unprovoked seizure, years | 0.98 (0.96 – 1.0) | 0.07 |
| Intercept | 0.31 |  |

**Abbreviations**: CI – confidence interval, EEG – electroencephalogram, MRI – magnetic resonance imaging

**Supporting information** **figure S1: Nested cross-validated performance of imaging-only and clinical-only models**


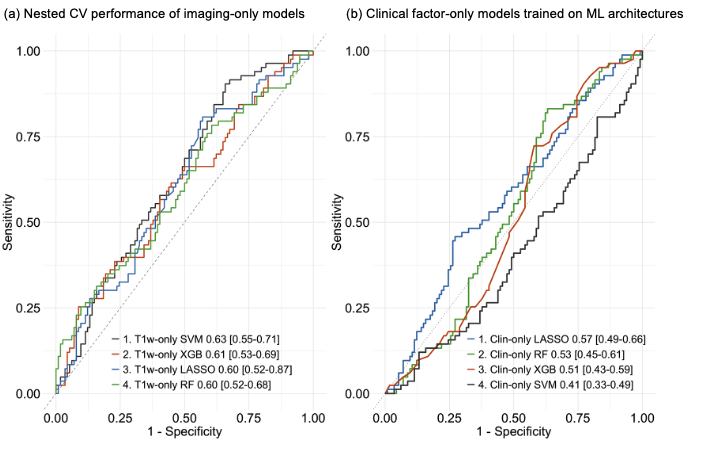


**
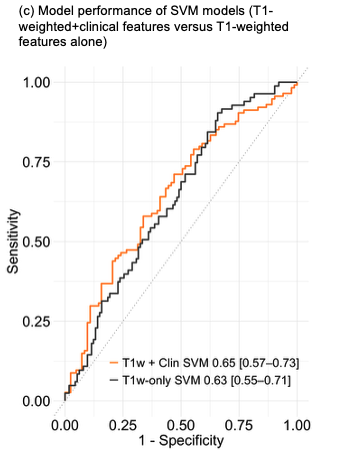
**

**(a) Nested cross validation model performance for models trained on T1-weighted imaging features only, showing the highest AUC [95% confidence interval] of each model algorithm class.** The radial kernel basis Support Vector Machine (SVM) with PCA variance filter was the best performing imaging-only model, followed by XGBoost with PCA variance filter, LASSO with PCA variance filter and random forest with feature importance filter

(b) **Model performance of clinical-only models trained on the same machine learning architectures;**

**(c) Model performance of SVM trained using imaging + clinical features and imaging features alone**

**Abbreviations**: ML – machine learning, SVM – support vector machine, RF – random forest, LASSO - Least Absolute Shrinkage and Selection Operator, XGB – eXtreme Gradient Boosting, T1w – T1-weighted imaging features, Clin – clinical feature-only model, AUC – area under the receiver operating characteristic curve, PCA – principle components analysis, CI – 95% confidence interval

**Supporting information** **figure S2: Calibration plot of Support Vector Machine model trained on combined clinical and imaging features**


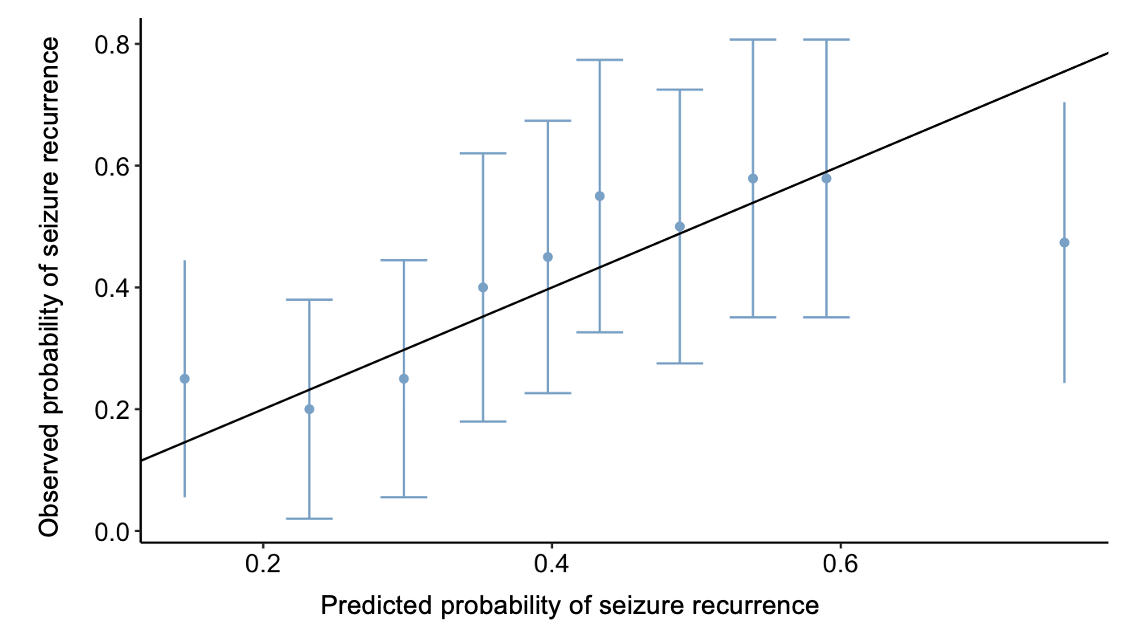


Calibration plot of the internally cross-validated Support Vector Machine (SVM), showing predicted versus observed probabilities of 12 month seizure recurrence. The black line represents perfect calibration. The blue vertical lines around each point represent confidence intervals for the observed proportion of events in that bin.

**Supporting information** **figure S3: Post-hoc group-level differences in imaging features with highest global feature importance**


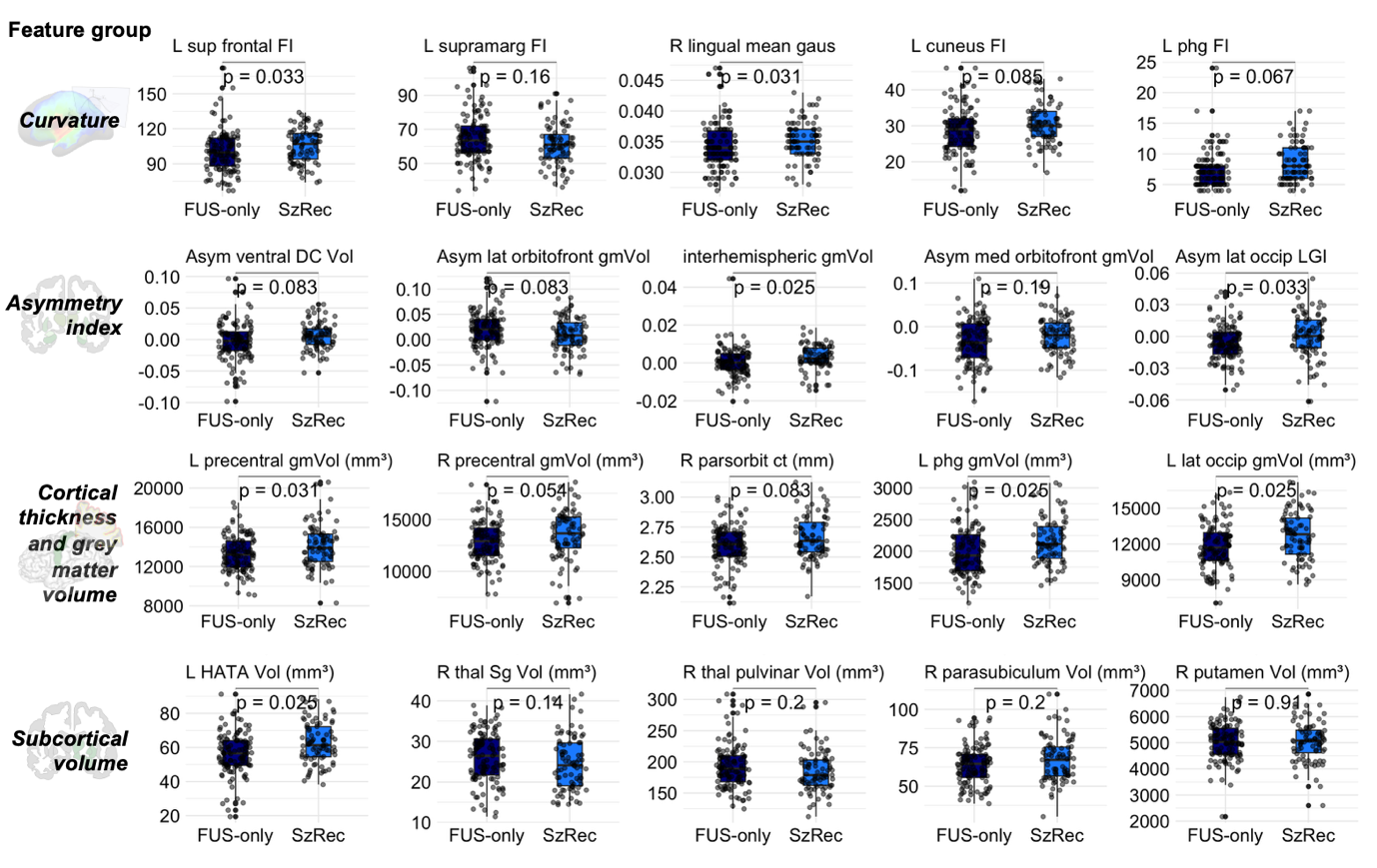


Boxplots show the distribution of each of the top 20 imaging features based on global feature importance (absolute mean Shapley value) for individuals with first unprovoked seizure (FUS)-only versus seizure recurrence. Statistical significance was assessed using two-sided Wilcoxon rank-sum tests for each feature, with p-values corrected for multiple comparisons using the Benjamini–Hochberg false discovery rate (FDR) method. Features with corrected p < 0.05 are considered statistically significant. Note that these analyses were performed post hoc after model training and were not used to inform feature selection or model development.

**Abbreviations**: FUS-only – first unprovoked seizure only and SzRec – seizure recurrence at 12 months follow-up, L – left, R – right, FI – folding index, mean gaus – mean Gaussian curvature, phg – parahippocampal gyrus, Asym – left/right hemispheric asymmetry, ct – cortical thickness, sup – superior, supramarg – supramarginal, Vol – volume, DC – diencephalon, lat – lateral, med – medial, orbitofront – orbitofrontal, occip – occipital, gmVol – grey matter volume, LGI – local gyrification index, parsorbit – parsorbitalis, thal pulvinar – pulvinar subnuclei of thalamus, thal Sg – suprageniculate nucleus of thalamus, HATA – hippocampal-amygdala transition area
